# Three-dimensional virtual surgery models for percutaneous coronary intervention (PCI) optimization strategies

**DOI:** 10.1038/srep10945

**Published:** 2015-06-04

**Authors:** Hujun Wang, Jinghua Liu, Xu Zheng, Xiaohui Rong, Xuwei Zheng, Hongyu Peng, Zhanghua Silber-Li, Mujun Li, Liyu Liu

**Affiliations:** 1 Department of Precision Machinery and Precision Instrumentation, University of Science and Technology of China, Hefei 230026, China; 2 Key Laboratory of Soft Matter Physics, Institute of Physics, Chinese Academy of Sciences, Beijing 100190, China; 3 Department of Cardiology, Beijing Anzhen Hospital, Capital Medical University, Beijing Institute of Heart, Lung and Blood Vessel Disease, Beijing 100029, China; 4 State key laboratory of Nonlinear Mechanics, Institute of Mechanics, Chinese Academy of Sciences, Beijing 100190, China

## Abstract

Percutaneous coronary intervention (PCI), especially coronary stent implantation, has been shown to be an effective treatment for coronary artery disease. However, in-stent restenosis is one of the longstanding unsolvable problems following PCI. Although stents implanted inside narrowed vessels recover normal flux of blood flows, they instantaneously change the wall shear stress (WSS) distribution on the vessel surface. Improper stent implantation positions bring high possibilities of restenosis as it enlarges the low WSS regions and subsequently stimulates more epithelial cell outgrowth on vessel walls. To optimize the stent position for lowering the risk of restenosis, we successfully established a digital three-dimensional (3-D) model based on a real clinical coronary artery and analysed the optimal stenting strategies by computational simulation. Via microfabrication and 3-D printing technology, the digital model was also converted into *in vitro* microfluidic models with 3-D micro channels. Simultaneously, physicians placed real stents inside them; i.e., they performed “virtual surgeries”. The hydrodynamic experimental results showed that the microfluidic models highly inosculated the simulations. Therefore, our study not only demonstrated that the half-cross stenting strategy could maximally reduce restenosis risks but also indicated that 3-D printing combined with clinical image reconstruction is a promising method for future angiocardiopathy research.

Coronary artery disease, which commonly results from severely narrow coronary atherosclerotic lesions, is one of the most lethal diseases globally^1^. In the entire coronary artery tree, bifurcation sites are prone to develop atherosclerotic plaque because of turbulent blood flow. Similarly, restenosis is more likely to occur in this area after treatment with stent implantation. Increasing clinical evidence has demonstrated the default strategy for treating bifurcation lesions was a single stent implantation in the main vessel with provisional stenting to the side branches.

However, there is no consensus on detailed implantation locations for single stents in bifurcation lesions, especially for some bifurcation subtypes. For example, non-aorto ostial coronary stenosis is a relatively rare and special bifurcation lesion in which only one side branch ostium is involved (i.e., Medina classification 0, 0, 1 or 0, 1, 0)^2^. Several alternative single stenting strategies, namely “stent implantation positioning”, which includes precise ostial stenting and crossover stenting that covers the branch ostium, have been proposed for the treatment of this subtype^3^,^4^. Fig. 1 shows a coronary angiogram that was provided by Beijing Anzhen Hospital. Fig. 1A shows the narrowed coronary artery with ostial stenosis (Medina 0, 1, 0 bifurcation lesion) was opened with precise ostial stenting. However, one year later, as shown in Fig. 1B, in-stent restenosis occurred, which is indicated by the red arrows in the figure. Characterizing optimal stent positioning in this bifurcation subtype is of interest to cardiologists. Unfortunately, few clinical trials have been performed for this evaluation, probably due to the relatively low prevalence of this lesion and small number of samples. Thus, the preferred treatment option is still in question.

Research is required to improve treatment for these stenting problems. Historically, studies have indicated that different stenting strategies could result in different distributions and low shear stress areas in bifurcation sections^5^,^6^. The altered local hemodynamic profile caused by a stent could be associated with subsequent stent failures (e.g., in-stent restenosis and stent thrombosis), which remain clinically significant problems^5^,^7^,^8^. In detail, two major factors are responsible for restenosis. First, low wall shear stress (WSS) is closely related to the occurrence of stenosis, in-stent restenosis and stent thrombosis, as reported previously. Fig. 1C shows when a stent is placed inside the vessel, the metallic structure generates localized high WSS and low WSS regions simultaneously because of its non-negligible thickness (~100 μm). The high WSS on top of the stent activates platelets to release adenosine diphosphate (ADP), a potent platelet aggregation promoter. At the same time, recirculation regions with low WSS downstream of the stent increase local concentrations of activated platelets, retard re-endothelialization, and attenuate the production of natural anticoagulants, such as nitric oxide (NO), prostacyclin (PGI2) and tissue plasminogen activator (tPA)^8^,^9^,^10^,^11^. It has been shown that low WSS (≤0.4 Pa), which is prevalent at atherosclerosis-prone sites, stimulates an atherogenic phenotype^9^,^12^. Therefore, improper stent positions could bring unnecessarily large low WSS regions and increase restenosis possibilities. In addition to the localized low WSS, stent implantation could also result in hydrodynamic changes inside the vessel, generating excessive low WSS at distant sites and cause thrombogenicity. These sites are mostly sensitive to the stent position, as indicated by the blue circles in Fig. 1D. In summary, to avoid the thrombogenicity process and to lower the risks of restenosis after percutaneous coronary intervention (PCI), our research focused on studying optimized stent implantation positions and ideal treatment strategies for non-aorto ostial lesions that could minimize localized low WSS areas as well as reduce low WSS sites.

In this study, we explored optimized stent implantation strategies in the clinic through both simulation and experimental approaches. In the past, hemodynamic studies on coronary bifurcation were mostly performed with a computational fluid dynamics (CFD) method on ideal models, such as typical “Y” shaped simulated vessels^12^,^13^,^14^. Our study provided a more realistic approach and reconstructed a vessel digital model from a clinic case. The CFD and related analysis produced a relatively optimized stent position. Moreover, the model was successfully constructed in *in vitro* settings through 3-D printing technology. With the help of microfabrication, microfluidic chips implanted with real stents were achieved to mimic PCI surgery *in vivo*. Hydrodynamic experiments demonstrated that the 3-D microfluidic models were highly consistent with the simulated results and the 3-D printing model is a promising method for future coronary disease studies.

## Materials and methods

The study used a clinical case in which restenosis occurred one year after PCI. Both CFD simulation and *in vitro* microfluidic experiments were performed to study the flow field and its characteristics affected by the stent.

The *in vitro* study was performed as the following. First, a clinical 3-D digital vessel model was generated by angiography image processing and reconstruction. Then, the digital model was created with a 3-D printer and an *in vitro* wax model was realized. It was later duplicated into identical polydimethylsiloxane (PDMS) chips with real stents implanted. Subsequently, microfluidic and hydrodynamic experiments were performed with real-time visualization.

**Figure 1:**
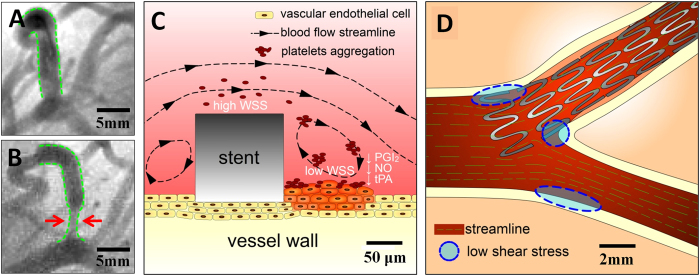
(**A)** A clinic angiography image from the Anzhen Hospital shows that a branch vessel with stenosis has been expanded by a stent right after PCI; (**B**) Restenosis occurred at the stent position one year after PCI, as is indicated by the red arrows; (**C**) The cartoon image shows stent thrombogenicity. The stent thickness introduced low WSS, which facilitated outgrowths of epithelial cells and led to restenosis; (**D**) The stent positioning could have also affected the low WSS region area near the stent. The blue regions are possible restenosis sites related to the stent position.

3-D image reconstruction. A coronary angiogram (Fig. 1A) was produced with a single C-arm angiography system (AIXIOM Artis DFC, Siemens, Germany) in the hospital and processed into the 3-D vessel model by software (Solidworks 2012). The process included three steps.

**Figure 2:**
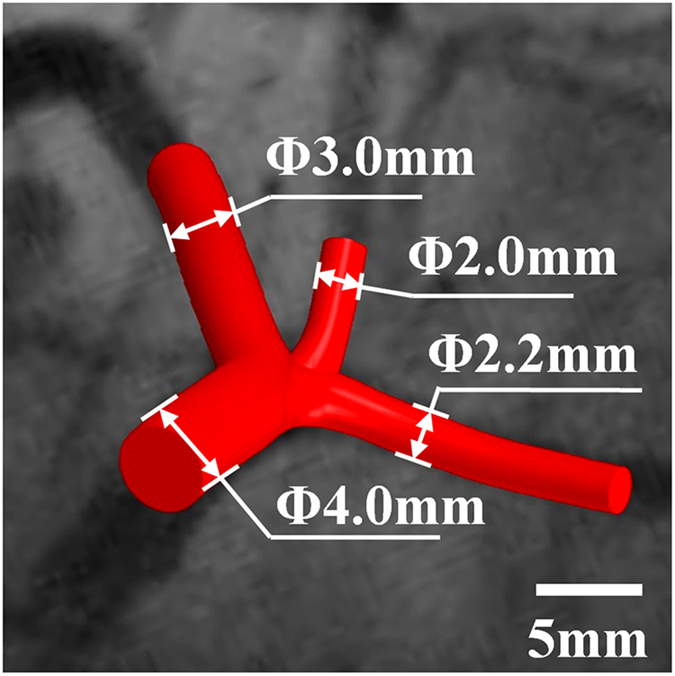


First, two angiography images at the same phase were placed in virtual space at specific angles that were recorded by the C-arm angiography system. The invasive coronary angiography included a series of dynamic images recorded during several seconds. The frames at the end of diastole were selected for image reconstruction. Then, the vessel centerlines were produced based on the projections of the two angiogram photos set previously. For better model reconstruction, the angle between the two images was more than 30°. Third, the vessel diameter was denoted with circles along the centerline and these circles were then linked together to compose the vessel wall. The last two steps were repeated several times to generate the most precise model^15^,^16^,^17^. Fig. 2 shows the original digital vessel model (without a stent), which had one main branch (D = 4.0 mm) and three sub-branches (D = 3.0 mm, D = 2.2 mm and D = 2.0 mm). Fig. 2 shows the digital model highly inosculated the clinic angiography results.

### CFD simulation for PCI strategies

**Figure 3:**
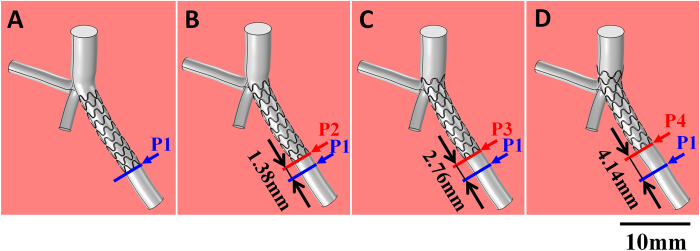
Four PCI strategies are illustrated with stents implanted at different positions in subbranches. (**A**) the ostial stenting strategy; (**B**) the half-cover strategy; (**C**) and (**D**) are crossover strategies. The vertical branch is the main branch.

After the digital vessel model was created, it was utilized for CFD analysis of stenting strategies and related effects. The stent was composed of mesh structures (15.0 mm long with a diameter of 100 μm) and was constructed digitally following the stents used in the clinic. Four stent spatial positions were displayed to simulate four surgical strategies in PCI, as is shown in Fig. 3.

Strategy I is the ostial stenting strategy where the stent top end stays in the original stenosis upper area. In this case, the stent was completely located inside the sub-branch. Each time the stent position was raised 1.38 mm upward along the branch, the strategy changed into strategy II, strategy III, or strategy IV step by step. Strategy II is a half-cover strategy where the stent top end is in the middle of the bifurcation region. Strategies III and IV are called crossover strategies. In strategy III, the stent top end slightly contacted the main branch wall and in strategy IV the stent top end was inserted into the main branch. The four strategies represent typical instances in actual PCI surgeries.

Based on the models above, COMSOL Multiphysics (version 4.2) was used to simulate blood flow in the vessel. The blood density was set as 1.060 × 10^3^  kg/m^3^, and its viscosity was 3.5 × 10^−3^ Pa·s. The blood was assumed to be a Newtonian incompressible fluid. Because the Reynolds number (*Re*) was approximately 48, it was a laminar flow (*Re* < 1000) and the *Re* was defined as:


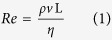


where *ρ* is the density of the liquid and *μ* is the viscosity; *L* and *v* are the typical size and the velocity of the fluid flow, respectively.

The blood flow followed the continuity equation and Navier-Stokes equations:


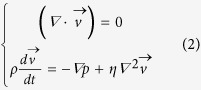


Here *p* is the pressure, 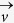 is the blood flow velocity, *ρ* is blood density and *η* is the viscosity of the blood flow^18^,^19^,^20^. The fluid computation and simulation were performed based on these governing equations. For the inlet boundary condition, the inlet flow rate was set at 60 mL/min, which is the average flow rate of the left coronary artery for a normal adult at rest^13^,^21^. Additionally, the rigid vessel wall and a no-slip condition were applied to the walls^18^,^19^,^20^,^21^. For the outlets, the downstream microcirculation resistance was considered and Murray’s law was used to estimate the boundary conditions. More details about the outlet boundary conditions are provided in the supplementary methods S1.

Furthermore, the simulation results offered velocity fields in the vessel and the WSS, or endothelial shear stress, τ, could be calculated by obtaining the velocity gradient:


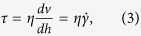


where *h* is the distance to the vessel wall, and 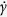 is the wall shear rate. The unit of the shear stress was N/m^2^ or Pa and that of the shear rate was s^−1^.

### 3-D printing for an *in vitro* microfluidic model

**Figure 4:**
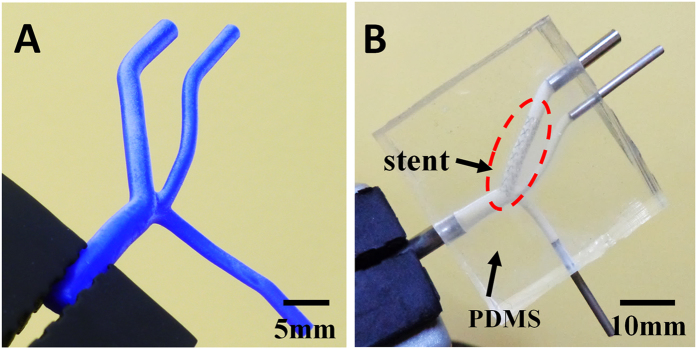
(**A**) The 3-D wax model was fabricated with a 3-D printer (HD3500, 3D Systems); (**B**) The wax model was used as a positive mold and was used later to fabricate the PDMS chip. Subsequently, a metallic stent was implanted into the sub-branch following Strategy II.

The 3-D model was made of a wax material (VisiJet^®^ Hi-Cast) and was printed with a commercial 3-D printer (ProJet^TM^ HD3500, 3D Systems, USA) at the highest resolution of the machine setting (16 μm), as is shown in Fig. 4A. At the beginning of the process, the printed model was placed in a petri-dish and a liquid PDMS (Dow Corning, USA) mixture was poured into the dish. After degassing in a vacuum chamber for approximately 10 minutes, the device was levelled for 72 hours at room temperature until the PDMS solidified. Subsequently, the PDMS solid block with 3-D structures was cut into a square and the ends of the wax vessels were exposed externally. When the block was immersed in liquid n-hexane in an ultrasonic bath, the wax dissolved gradually. The ultrasonic cleaning process was refreshed in n-hexane five times. Each refreshment period lasted 10 minutes to prevent penetration of the melted wax into the PDMS. Finally, the PDMS chip was rinsed by de-ionized water and dried by nitrogen gas until the channels inside the PDMS block became transparent.

The stent (Tivoli, Inc.) implanted inside the PDMS channel was 15.0 mm in length and approximately 3.0 mm in diameter after expansion. The implantation process; e.g., the “virtual surgery”, consisted of the following procedures. First, the channels were filled with water so they had enough transparency for stent observation. Then, with the help of a physician, the stent was inserted inside the channel under an inverted microscope (Ti, Nikon) and reached the exact position required for strategy II. The external controlling balloon expanded the internal stent at 12 atm and made it spontaneously fit the walls of the channel. After the stent was fixed inside the chip, its supporting wire was extracted, leaving a solo stent inside. Finally, four stainless tubes were installed at each channel inlet to be connected to syringe pumps later. Fig. 4B shows the complete chip with the stent position labelled by the red circle.

**Figure 5:**
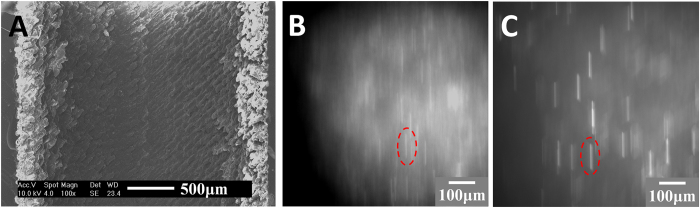
(**A**) The SEM image shows that the internal surface of the PDMS channel had snakeskin-like roughness; (**B**) and (**C**) show the trajectories of fluorescent beads mixed in water and glycerine-alcohol solution when flowing inside the channel, respectively. The beads highlighted by the red circles were at approximately 3.0 × 10^−3^ m/s and the bead in (**C**) shows a clearer image of its trajectory. Fig. 5B is not clear mainly due to the difference between the water refractive index and the PDMS refractive index. To overcome this difficulty, we developed a new method, which is described in detail in the section “Artificial blood preparation”.

Artificial blood preparation. Channel transparency in the PDMS microfluidic chip is key for observation and analysis in a hydrodynamic study. To analyze its inner smoothness, a channel in a PDMS chip was cross-sectioned and characterized by a scanning electron microscope (SEM; XL30S-FEG, FEI, U.S.A). The SEM picture (Fig. 5A) demonstrated that the inner surface of the PDMS channel exhibited snakeskin-like roughness. This indicated that, although the commercialized 3-D printer from 3-D Systems reached the highest resolution of its kind, the printed structures still had unsatisfying resolution. To test the influence on the following observations, the following procedure was carried out. De-ionized (DI) water was mixed with fluorescent beads (Thermo Scientific, 2 μm in diameter) with a density of 1.05 × 10^3^ kg/m^3^ and excitation/emission wavelengths of 542 nm/612 nm, respectively. The mixture had approximately 20 μL fluorescent particles in 100 mL DI water. Fig. 5B shows the syringe pump (Chemyx Inc., Stafford, TX) with the injected mixture inside the channel, which was imaged by the microscope simultaneously. The image shows that the red circled bead, although showing visible fluorescent traces, had a blurred quality and was not acceptable for quantifying the flows in the channel. There were two major reasons for this. First, the snakeskin wall introduced multiple complex reflective surfaces. Moreover, the water refractive index (n = 1.332) had contrast with the PDMS refractive index (n = 1.406). The refraction at the water and PDMS interface seriously affected the homogeneity of light emission and significantly lowered the imaging quality. Although the resolution of the 3-D printed structures could not be improved at this stage, in order to solve this problem, a solution of glycerine and alcohol was used in replace of DI water. The experiment showed that the solution index could be altered by adjusting the mixture ratio. When glycerine was mixed with alcohol at a 9:11 mass ratio, its refractive index was approximately 1.403, which was the same as that of PDMS^22^. The density of the solution was 0.96 × 10^3^ kg/m^3^, and the viscosity was 8.06 × 10^−3^ Pa·s, which were measured with a Pinkevitch viscometer at 25 °C. Subsequently, the fluorescent beads were mixed inside at the same ratio as that mixed in water previously (1:5 × 10^3^ in volume ratio). Fig. 5C shows that the new solution, as artificial blood, significantly improved the image quality and the trajectories of the fluorescent particles in the channels had clear visualization. The specific particle velocity could then be quantified. For example, the particle circled in red had a velocity of approximately 3.0 × 10^−3^ m/s.

*In vitro* microfluidic experiment. First, a glycerine-alcohol solution without fluorescent particles was used to pre-fill the chips one hour before the experiment started. The chips were then put into the vacuum chamber to remove bubbles. During the experiment, three syringe pumps controlled the flow of the solution with fluorescent particles inside the channels, leaving one free outlet that could adjust its boundary condition automatically.

**Figure 6:**
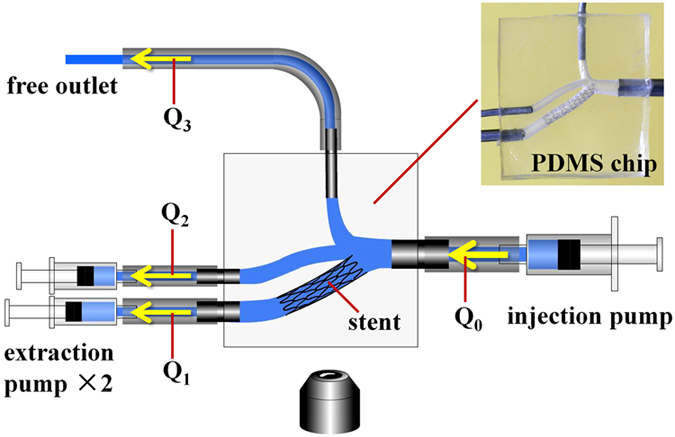
The microfluidic chip was connected with and controlled by the external syringe pumps. The setup simulates the vascular system and is imaged simultaneously with inverted microscope.

Fig. 6 shows the setup in detail. The flow rate was set at 60 mL/min at the main branch (*D*_*0*_ = 4.0 mm). The value represented the average blood flow rate *in vivo* and was the same as the inlet boundary condition in the CFD simulation. The other two syringe pumps were connected to two of the outlet branches for inhaling the solution. The flow rates were determined by Murray’s law (*Q*∝*D*_i_^3^, see supplementary methods S1 for details) and set at 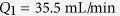
(for outlet branch 1, *D*_1_ = 3.0 mm) and 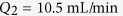
(for outlet branch 2, *D*_2_ = 2.0 mm). Automatically, the flow rate of the free outlet branch 3 was 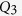 = 14.0 mL/min (for outlet branch 3, *D*_3_ = 2.2 mm) based on mass conservation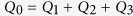. After that, the inverted microscope was employed to visualize fluorescent particle trajectories at interesting spots. The velocity and WSS were calculated and analyzed thereafter to characterize the flow fields.

Next, observation was performed with an inverted microscope (Olympus IX71) with a 10x/NA = 0.3 objective in a 25 °C environment. By adjusting the objective focal plane, clear visions of the main branch were found in a bright field. Subsequently, bead fluorescent trajectories were detected and recorded by an EMCCD (iXon Ultra 897, Andor). The exposure time was 1.5 ms, and the time interval was 17 ms between two successive frames. The image had a 512 × 512 pixel resolution corresponding to an 800 × 800 μm field of view. The obtained images were processed by ImageJ, during which the particle trajectories were marked and their lengths and distances to the wall were measured. This procedure was similar to the method called micro-particle trajectory velocimetry (mirco-PTV), which is used in microflow measurements^23^,^24^ The length of the particle trajectory represented its displacement transported by the local flow in the exposure time Δt_exp_. The particle velocity was then calculated by dividing the trajectory length of the fluorescent particles by the exposure time. To obtain the velocity profile, we set some interrogation regions that were approximately 100 μm in length along the flow direction and approximately 30 μm in width normal to the wall surface. The average length, L_a_, of approximately 30 particle trajectories caught in the same interrogation region was calculated. Then, the velocity, u, at the center of the interrogation region was obtained by u = L_a_/Δt_exp_. The velocity profile was then obtained by knowing the velocity value of each individual interrogation region. Furthermore, presuming a linear velocity distribution very close to the vessel wall, the WSS, τ, was then calculated as: τ = ηu/z based on eq. (3), where z = 15 μm is the distance from the interrogation center to the wall.

## Results and discussion

### CFD simulation

**Figure 7:**
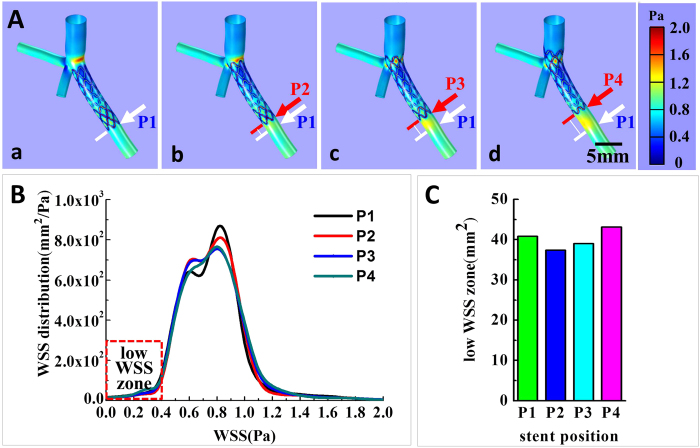
(**A**) The CFD simulation illustrates WSS distribution on the internal surface of vessel walls, which are in different stent positioning strategies from ostial stenting (**a**) to crossover (**d**) (**B**) The plotted WSS distribution of the four strategies. Precious study showed low WSS (≤0.4Pa) contributed to high restenosis risk, as is highlighted in the figure; (**C**) The summary and comparison of low WSS (≤0.4Pa) areas in the four stent positioning strategies. Strategy II was the best strategy with the smallest area of low WSS, and strategy IV has the largest area.

As stated previously, stent positioning is likely to affect the total area of low WSS and further raise the possibility of restenosis. Fig. 7A shows CFD analysis of the four typical stent positioning strategies in PCI. The main vessel branches were placed upwards, and the stent was allocated to the branch toward the lower right corner. The red arrows indicate the end of the stent and the white arrows indicate the initial position of the stent end in inset (a). The computed WSS distribution is demonstrated on the vessel surface with a color gradient change. Dark blue and light red represent low WSS and high WSS, respectively. The color bar shows that the WSS ranged from 0.0 Pa to 2.0 Pa.

Fig. 7B is the plot of the calculated WSS distribution on the vessel internal surface of all branches. Previous studies have suggested that hemodynamic shear stress is important for the determination of endothelial function and phenotype. The arterial level shear stress (>1.5 Pa) induces endothelial quiescence and an atheroprotective gene expression profile, while low shear stress (≤0.4 Pa), which is prevalent at atherosclerosis-prone sites, stimulates an atherogenic phenotype^9^,^12^. Therefore, areas of low WSS (WSS ≤0.4 Pa) are considered as the evaluation index in our strategy evaluation, as is highlighted by the red rectangular in Fig. 7B. The total low WSS area in each strategy indicates the risks of restenosis: the smaller the area is, the lower the possibility for restenosis and the better the strategy. Fig. 7C shows a summary of low WSS (≤0.4 Pa) areas. From strategy I to the strategy IV, the areas were 40.80 mm^2^, 37.37 mm^2^, 39.00 mm^2^ and 43.14 mm^2,^ respectively. We concluded that strategy II, which is called the half-cover strategy, had the fewest low WSS (WSS ≤ 0.4Pa) areas and may be a relatively better strategy among the four. The four strategies did not have significant differences. In another words, the low WSS (WSS ≤ 0.4Pa) areas of the best strategy II were only approximately 10% smaller than those of the strategy IV. Hence, strictly speaking, the suggested most optimized strategy could only lower, but not prevent, restenosis risk. However, in the clinic, a 10% possible risk would have an effect on patients because clogging is a chain reaction. A better strategy with 10% fewer low WSS areas could significantly decrease the possibility of subsequent restenosis.

**Figure 8:**
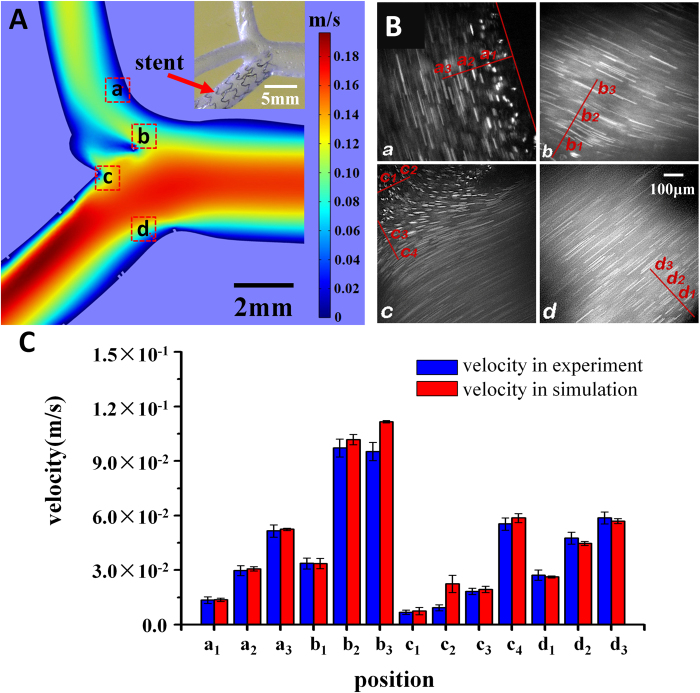
Correspondence between the CFD simulation and the microfluidic experiment. (**A**) The CFD simulation results showed that the velocity distribution along the middle cross-section of the main branch in the vessels when the stent was implanted with strategy II. Four regions of interest were selected for further analysis and comparison with the *in vitro* experiment. The upper-right inset shows the stent position inside the chip channel. (**B**) The fluid flow was imaged with an inverted microscope. The velocities at interrogation points in the regions that correspond to the simulation are labelled and measured; (**C**) The listed velocities demonstrated that the experiment and the simulation had a satisfactory coincidence.

After the most optimal strategy; i.e., strategy II, was determined, the CFD analyzed the stent effect on the flow field, as is shown in Fig. 8A. The image shows only three branches because the fourth was not in the same horizontal plane. The red color represents the positions with higher flow rates while the blue represents the positions with lower flow rates. It can be observed that the flow increases the rate from the main branch to the stent branch and the stent led to some corners obtaining a lower flow rate (in blue). The simulation had four typical square regions, indicated as regions (a)–(d), which are highlighted in red square frames. The results showed that region (a) and region (d) were most likely to be the low WSS areas with sharp variation in flow rates. The velocity distributions of region (b) and region (c) were altered by the stent structure.

### Microfluidic experiment

To test the feasibility of the *in vivo* microfluidic model, further hydrodynamic experimentation with the 3-D printed model was performed in the chip. It followed strategy II and had the stent placed inside the PDMS vessel, as is shown in the upper-right inset. Fig. 8B demonstrates the trajectories of fluorescent particles in the flux of the glycerine-alcohol solution. Corresponding to the simulation results, four regions at exactly the same spatial positions of the CFD model were found in the microfluidic channel and inside flow fields were imaged and analyzed. Each image was obtained by overlapping five successive slices together, and the detailed flow field of the selected spots are shown in Fig. 8A. Because trajectory length represents the bead velocity, the longer the length, the faster the velocity is. For example, inset (c) shows that the flow rate was slower in the upper-left corner but it increased dramatically in the lower-right directions. This field profile highly resembled the simulation results in region (c), which is shown in Fig. 8A. To quantify the flow field, in each inset, 3–4 interrogation positions that were close to the channel wall were selected and their velocities were calculated. The details of obtaining velocity profiles were introduced above in the “*in vitro* microfluidic experiment” section.

Figure. 8C shows a comparison of the specific interrogation positions (a_1_, a_2_…etc.) between the measured velocities in the *in vitro* experiment and the simulation results. Blue bars are the measured velocity from the *in vitro* experiment, and red bars are the velocities obtained from the CFD simulation. The interrogation position was prescribed in this way: based on the centered position, an area is defined that is 20 pixels (~30 μm) long and is approximately parallel to the wall. Approximately 20 trajectories were selected from this area and their average velocities with standard errors were calculated. The diagram demonstrates that the experiment and the simulation had satisfactory agreement, in which the average difference was only approximately 5%. Although viscosity and density of the artificial solution were not the same as those of human blood, further study has demonstrated that the solution could still mimic the flow field of human blood *in vivo* (see supplementary discussion S2).

The particle trajectories could help obtain not only the velocity distribution *in vitro* but also the WSS by equation (3). The WSS in both the simulation and experiment were calculated in the following way. The velocity of the spot that was closest to the vessel wall was divided by its distance to the vessel wall and then multiplied by the viscosity. Table 1 shows that the experimental WSS was divided by a constant 2.30, which was due to the variation of the viscosity between the artificial blood and real blood (*η*_exp_/*η*_CFD_* = *8.06* × *10^−3^ Pa·s /3.5 × 10^–3^ Pa·s = 2.30, based on equation [3]). Because region b was not adjacent to the vessel wall, the WSS was not calculated; region c had two results as it had two typical wall zones. The results showed that the experiment highly conformed to the simulation results with the variations ranging from approximately 2% to 7%. Moreover, as discussed previously, the typical low WSS related to the stenosis was ≤0.4 Pa; the results indicated that region A and region C were typical low WSS regions, while region D was not. These findings implied that the hemodynamic factors may not be the only cause of the in-stent restenosis and stent thrombosis, and the injury and pressure introduced by the stent on the vessel wall could also contribute to stent failures.

Not limited to the current results, *in vitro* microfluidic experiments are believed to have much potential for future coronary disease study. Currently, the refractivity of the artificial blood was the same as the PDMS, while the viscosity of the prepared fluid was 8.06 × 10^−3^Pa·s, which was higher than human blood (3.5 × 10^−3^ Pa·s). Although our analysis showed that the solution could highly simulate human blood flow profiles with a Reynolds number *Re* ~ O(10), future research will focus on discovering more appropriate solutions with a refractive index and viscosity closer to real blood. In addition, *in vivo* vessels are highly elastic and deformable; however, the current chips have rigid channels due to the PDMS fabrication procedures. Therefore, the study has not realized the process of the implanted stent automatically and spontaneously enlarging and re-shaping the plague region. Future efforts will focus on introducing *in vitro* thin and elastic vessels (approximately 3 mm in thickness) with updated microfabrication procedures and will achieve more realistic modelling. Moreover, elastic mechanics modelling will be considered in future CFD simulations. In addition, the vascular endothelial cells could be cultured and developed in channels, which would be a big step in approaching the *in vivo* vascular microenvironment as well as enabling the physiological and hemodynamic study of epithelial cell behaviors inside. The flows would also be pulse controlled externally to simulate the dynamic blood flows from the heart.

## Conclusions

In this report, we presented a novel approach for hemodynamic study of coronary disease and explored optimized stent positions in PCI. With the merit of microfluidics and 3-D printing technology, the research was performed not only in CFD simulation but also in *in vitro* hydrodynamics. Our study had three advantages. First, the study was based on a real and typical clinical example, and the study aimed to develop a realistic approach and provide better strategy for clinical use. Second, 3-D printing technology was utilized successfully to construct *in vitro* vessel structures. Third, real stents were implanted into the *in vitro* microfluidic model to experimentally test their positioning effect. It is believed that our proposed method is an encouraging beginning and, with future updated techniques, it will allow for further exciting angiocardiopathy studies.

## Additional Information

**How to cite this article**: Wang, H. *et al.* Three-dimensional virtual surgery models for percutaneous Coronary intervention (PCI) optimization strategies. *Sci. Rep.* **5**, 10945; doi: 10.1038/srep10945 (2015).

## Supplementary Material

Supplementary Information

## Figures and Tables

**Table 1:** The WSS calculated for the experiment and the simulation.

	WSS_EXP^a^(Pa)	WSS_CFD^b^**(Pa)**	variation^c^
**a**	0.387 ± 0.050	0.379	2.08%
**b**	—	—	—
**c**_**12**_	0.266 ± 0.045	0.281	5.59%
**c**_**34**_	0.676 ± 0.073	0.693	2.43%

^a^WSS calculated for the experiment based on equation (3)

^b^WSS calculated for the simulation results

^c^the difference between WSS_EXP and WSS_CFD
