# Supplementary material for: Three-dimensional virtual surgery models for percutaneous coronary intervention (PCI) optimization strategies
Source: Sci Rep. 2015 Jun 4;5:10945. doi: 10.1038/srep10945 (PMC4455241; doi:10.1038/srep10945)
Supplement: Supplementary Information [file srep10945-s1.pdf]

Three-dimensional virtual surgery models for percutaneous coronary intervention (PCI) optimization strategies

Hujun Wang[1,2,#], Jinghua Liu[3,#], Xu Zheng[4,#], Xiaohui Rong[2], Xuwei Zheng[3], Hongyu Peng[3], Zhanghua Silber-Li[4,*], Mujun Li[1] and Liyu Liu[2,*]

Supplementary methods S1

The outlet boundary conditions for CFD simulation. The outlet boundary conditions are more complicated than the inlet boundary condition. It is well known that the flow rate relationship follows Murray's law; i.e., $Q \propto r^k$, where r is the average radius of the vessel branch, and the value of k is 3 at most times. The quantity of the blood flow at the inlet Q_0 should equal the sum of the blood flow at outlets, which means that the blood flow should follow the law of mass conservation when the blood vessel wall is assumed to have no leakage. The outlet flow relationship in the normal condition (health vessels without stent and stenosis) is:

$$\begin{cases} Q_1 : Q_2 : Q_3 = r_1^3 : r_2^3 : r_3^3 \\ Q_0 = Q_1 + Q_2 + Q_3 \end{cases} \quad \text{(S1)}$$

When there is stenosis or any type of obstruction, the flow rate relationship will not follow Murray's law. While it could be used for calculating the downstream microcirculation resistance, Murray's law should not be used for setting the outlet boundary condition directly. Murray's law implies that $R \propto r^{-3}$, or that the resistance to flow of each branch is inversely related to vessel size, with the same exponent k.[20] The total resistance of the blood, including the downstream microcirculation resistance, R_{tot}, is calculated by the equation:

$$R_{tot} = \frac{p_0}{Q_0} \quad \text{(S2)}$$

Q_0 (=60 mL/min) is the inlet flow rate at the main branch, p_0 (=100 mmHg) is the average blood pressure in the artery. The pressure and outflow distances are set as the outlet boundary conditions. The outflow is assumed as laminar and the pressure becomes 0 Pa at the distance behind the outlet.

The distance is calculated by the equation:

$$R_i = \frac{p_0}{Q_i} = \frac{p_0 \cdot \sum_{j=1}^{n} r_j^3}{Q_0 \cdot r_i^3} = \frac{8\eta l_i}{\pi r_i^4} \quad \text{(S3)}$$

R_i is the flow resistance of the blood flow (Poiseuille flow), η is the viscosity, r_i is the radius of the vessel branch, l_i is the outlet distance. Here, R_i of Poiseuille flow is prescribed at the outlet boundary to simulate the downstream microcirculation resistance.

Supplementary discussion S2

The feasibility of simulating blood flow field with a glycerine-alcohol solution

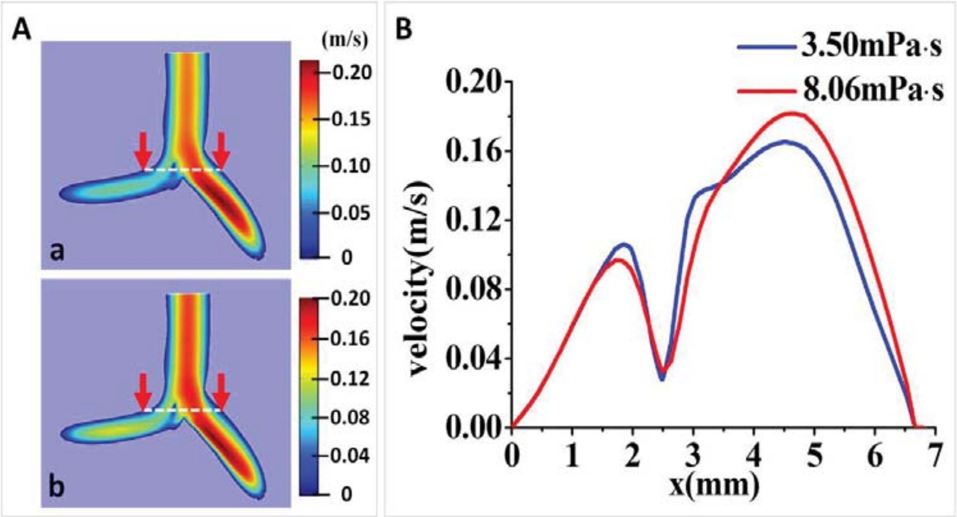

ESI Figure 1: (A) The CFD simulation results of the velocity distribution along the white dashed lines in the middle plane of the main branch vessel. The viscosity for (a) and (b) are 8.06×10-3 Pa·s and 3.5×10-3 Pa·s, respectively; (B) The velocity of the white dashed lines in two viscosities are plotted with positions. The velocity distributions are in high superposition, which implies that use of glycerine-alcohol is a good approach to test blood flow experimentally.

The viscosity and density of the glycerine-alcohol solution (artificial blood) are different from those of human blood, which raised the question whether the mircofluidic experiment could analyze the flow pattern *in vivo* as well as verify the rationality of the CFD simulation. The Reynolds number (*Re*) of the blood flow was estimated, which is a key dimensionless physical parameter to determine the flow characteristics. Here, the Reynolds numbers of the two viscosities were *Re*=19.1 (artificial

blood, $\eta=8.06\times10^{-3}$ Pa·s) and *Re*=48.5 (human blood, $\eta=3.5\times10^{-3}$ Pa·s) with the inlet flow rate of 60 mL/min. As the *Re* numbers were close to each other and were smaller than 1,000, they indicate laminar flows and their flow patterns could be similar. For further examination, CFD simulation was used to compare the velocity distribution of two different viscosity values, as shown in ESI Fig. 1A. The velocity distributions of the flows nearby the bifurcation, as indicated by the white dashed lines, were obtained and plotted with positions in ESI Fig. 1B. It can be concluded that the velocities at identical positions had high similarities. The results indicated that the artificial blood, though with a higher viscosity in the *in vitro* experiment, could still provide a reasonable simulation of blood flow in the vessel when *Re* ~ O(10).
